# The effects of menstrual cycle phase on jumping performance in women's football players: a systematic review

**DOI:** 10.3389/fspor.2026.1789611

**Published:** 2026-05-29

**Authors:** Catarina Marques, Miguel Rebelo, João Serrano, Hélder Fonseca

**Affiliations:** 1 Research Centre in Physical Activity, Health and Leisure (CIAFEL), Faculty of Sport, University of Porto (FADE-UP), Porto, Portugal; 2 Department of Sports and Well-Being, Polytechnic Institute of Castelo Branco, Castelo Branco, Portugal; 3 SPRINT - Sport Physical Activity and Health Research & Innovation Center, Castelo Branco, Portugal

**Keywords:** female athletes, football, hormonal fluctuations, oestrogen, progesterone, vertical jump

## Abstract

**Objectives:**

This systematic review aimed to evaluate the available evidence on the influence of menstrual cycle (MC) phases on vertical jump performance (countermovement jump and squat jump height) in female football players.

**Design:**

The review was conducted in accordance with PRISMA guidelines and registered in PROSPERO (ID1035357).

**Methods:**

A systematic search was performed in PubMed, Scopus, and SPORTDiscus (EBSCO). Studies were included if they involved female football players, assessed vertical jump performance, and compared performance across MC phases. Methodological quality was assessed using a modified Downs & Black checklist for non-interventional studies.

**Results:**

Seven studies met the inclusion criteria. Substantial heterogeneity was observed regarding MC phase determination procedures, sample characteristics, and testing protocols. Although some studies reported higher vertical jump height values during the follicular or ovulatory phases, no statistically significant differences were found.

**Conclusions:**

Current evidence is insufficient to determine whether MC phases influence vertical jump performance in female football players. Findings should be interpreted cautiously due to methodological limitations in MC phase determination and low statistical power. Future research should aim to standardize protocols, use objective hormonal verification, and recruit larger samples.

**Systematic Review Registration:**

https://www.crd.york.ac.uk/PROSPERO/view/CRD420251035357, PROSPERO: CRD420251035357.

## Introduction

In recent decades, women's football (soccer) has experienced substantial growth, driven by increased participation and advances in training methodologies tailored to female athletes. These developments include targeted strength and conditioning interventions aimed at improving physical fitness, muscular power, sprint ability, and injury prevention (1–5). It is estimated that around 29 million women play football worldwide, representing approximately 10% of all registered players (1).

In parallel, scientific interest in female-specific aspects of sports performance has increased, particularly regarding the influence of the menstrual cycle (MC) on training adaptations, injury risk, and physiological responses to exercise (6, 7). Despite this growing attention, women remain underrepresented in sports science research, accounting for only about 6% of studies in this field (8, 9).

Although women's football is becoming increasingly professionalised (10–12), research addressing menstrual health in female players remains limited. In particular, the potential impact of hormonal fluctuations across the MC on physical performance has not been sufficiently explored, especially regarding lower limb explosive actions such as vertical jumping (1).

The MC is a physiological process characterized by cyclic hormonal fluctuations over an average duration of 28 days (13, 14). It is typically divided into three phases: follicular, ovulatory and luteal. The follicular phase lasts approximately 12–14 days and is characterized by rising oestrogen and low progesterone concentrations. The ovulatory phase is shorter (1–3 days) and marked by a peak in oestrogen levels. The luteal phase, lasting around 12–14 days, is characterized by elevated progesterone and moderate oestrogen concentrations (14–18).

These hormonal fluctuations may influence connective tissue properties (19) and skeletal muscle function (14, 20). For instance, oestrogen has been linked to neuromuscular modulation and was suggested to affect force production (21), whereas progesterone has been associated with increased core temperature, perceived fatigue and reduced neuromuscular efficiency (17, 22). Consequently, some authors suggest that neuromuscular performance may be enhanced during the ovulatory phase and potentially reduced during the luteal phase (21).

In elite women's football, players cover approximately 10 km per match and perform frequent high-intensity actions requiring both aerobic capacity and muscular power (23). Lower limb strength and power are particularly important for actions such as sprinting, jumping, and shooting (6, 24). In this context, the Countermovement Jump (CMJ) is widely used to assess neuromuscular performance and lower limb muscle power, serving also as a practical tool for monitoring fatigue, detecting limb functional asymmetries and performance adaptations (3, 25–28).

Despite its relevance, the influence of MC phases on jumping performance in female football players remains unclear. Existing studies report inconsistent findings (14), with some showing no significant differences across phases (29–31), while others suggest phase related variations (32, 33). These discrepancies may also be explained by methodological differences, including approaches used to determine MC phases, sample characteristics, and testing protocols (29, 34). Additionally, menstrual disturbances such as anovulatory cycles or menorrhagia may further influence performance through altered hormonal profiles, increased fatigue and impaired recovery (35).

Therefore, the aim of this systematic review was to evaluate the available scientific evidence on the influence of MC phases on jumping performance in female football players.

## Methods

### Study design

This systematic review was conducted in accordance with the Preferred Reporting Items for Systematic Reviews and Meta-Analyses (PRISMA) guidelines (36). The protocol was pre-registered in the International Prospective Register of Systematic Reviews (PROSPERO: ID1035357).

### Search strategy

The search was conducted in the PubMed, Scopus and SPORTDiscus (EBSCO) databases. Studies published in English between March 2015 and March 2025 were included. This time restriction was applied to prioritize the inclusion of more recent data in order to reflect current research practices and improve the applicability of the results to current training and performance contexts, especially given recent advances in MC monitoring methodologies. However, it is acknowledged that this criterion may have excluded relevant previous studies.

The search strategy combined terms from the Medical Subject Headings (MeSH) and keywords related to the MC, neuromuscular performance and football. Boolean operators (“AND”, “OR”) were used to combine the terms as appropriate. The full search strategies for each database can be found in Supplementary Material S1. In addition, a snowball strategy was applied through manual screening of the reference lists of all included articles.

The final search was conducted on 25 March 2025.

### Eligibility criteria

The eligibility criteria were defined based on the PECOS strategy (Participants, Exposure, Comparison, Outcomes and Study Design) (Table 1). Vertical jump height (CMJ and SJ) was the only outcome considered in this review, as it was the most consistently reported and comparable measure across studies. Other neuromuscular variables were not consistently reported across studies and were therefore not included in the analysis. Studies were excluded if: (1) they included transgender athletes or participants using hormonal contraceptives; (2) lack of vertical jumping height through the CMJ or squat jump (SJ); (3) lack of specific data relating to MC phases; (4) not including women's football players in the sample; (5) theoretical studies, narrative reviews and grey literature (conference abstracts, dissertations or academic theses).

**Table 1:** PECOS eligibility criteria.

PECOS	Detail
Participants	Women’s football/soccer players of any competitive level
Exposure	MC phases (follicular, ovulatory or luteal).
Comparisons	Comparison of vertical jump height between the different MC phases
Outcomes	Vertical jump height measured through CMJ and/or SJ
Study design	Quasi-experimental, cohort, cross-sectional, descriptive and observational studies

### Study selection

Two reviewers independently conducted the study selection process in accordance with predefined eligibility criteria. All identified records were first exported to EndNote 21 (Clarivate Analytics, Philadelphia, PA, USA) to remove duplicates and then imported into Rayyan (https://www.rayyan.ai/, accessed 30 April 2025), an online platform designed to support the screening of systematic reviews.

Titles and abstracts were independently screened by both reviewers, followed by an assessment of the full text of potentially relevant studies. The PECOS framework was applied throughout the screening process. In addition, the reference lists of all included studies were manually searched to identify any additional relevant articles. Disagreements between reviewers were resolved through discussion, and a third reviewer was available to arbitrate if consensus could not be reached.

### Data extraction

Two reviewers independently extracted data from the included studies using a standardised template in Microsoft Excel. The information extracted included: study characteristics (authors and year of publication), participant details (sample size and competitive level), phases of the MC assessed (as reported in the original study), methods used to determine MC phase, outcome measures related to jumping performance (e.g., CMJ and SJ variables) and main conclusions.

The phases of the MC were grouped into three main categories (follicular, ovulatory, and luteal) to facilitate comparison across studies (14). When subphases were reported (e.g., early follicular phase, late follicular phase, or mid-luteal phase), these were assigned to the corresponding main phase. The harmonization approach and the terminology used are presented in Table 2.

**Table 2:** Standardization of terminology related to the phases of the MC in the included studies.

Standardized phase (used in this review)	Abbreviation	Terms reported in included studies
Follicular phase	FP	Early follicular phase (EFP); menstrual phase (MP); follicular phase (FP)
Ovulatory phase	OP	Late follicular phase (LFP); ovulatory phase (OP)
Luteal phase	LP	Mid luteal phase (MLP); luteal phase (LP)

To improve consistency across studies, competitive levels were classified as elite, sub-elite, under-17 or regional/amateur, based on the descriptions provided in each study. When studies reported multiple outcome measures, priority was given to jump height, as this is the most frequently used measure, is directly comparable, and is practically relevant for evaluating vertical jump performance across different studies (26).

### Methodological quality assessment

The methodological quality of the included studies was independently evaluated by two reviewers, using a modified version of the Downs and Black checklist (37). This scale was selected because it has been widely used in systematic reviews of non-interventional studies and allows for the assessment of key methodological domains relevant to observational and cross-sectional designs, including reporting quality, external validity, and internal validity (bias and confounding factors) (9, 38, 39). Twelve items were selected from the original 27-item scale, as they were considered the most appropriate for the types of studies included. More specifically, items related to randomization, blinding, and intervention procedures were excluded due to their lack of applicability to observational and cross-sectional studies, while items related to reporting quality, external validity, and internal validity were retained (Supplementary Material S2 for item descriptions and scoring criteria).

The selected items assessed key methodological domains, including reporting quality (items 1–4, 6–7 and 10), external validity (items 11–12), and internal validity related to bias and confounding (items 16, 18 and 20). Items relating to randomisation, blinding and intervention procedures were not considered due to their lack of applicability to the study designs included in this review.

Each item was scored as 1 (criterion met) or 0 (criterion not met or insufficiently described), resulting in a maximum possible score of 12. Studies were classified as having high methodological quality (10–12), moderate (7–9), low (4–6) or very low (0–3). Any discrepancies between reviewers were resolved through discussion, with a third reviewer consulted whenever necessary for arbitration. No studies were excluded on the basis of methodological quality.

Although Downs and Black's modified checklist provides a structured assessment of methodological quality, it has some limitations. In particular, it may not fully capture domain-specific sources of bias relevant to research on the MC, such as the accuracy of menstrual phase determination, small sample sizes, and limited control of confounding variables.

### Statistical analysis

A meta-analysis was not performed due to substantial heterogeneity among the included studies, particularly regarding study designs, methods used to determine MC phase, and outcome measures and respective testing protocols, which precluded a meaningful quantitative synthesis. Consequently, a structured narrative synthesis was conducted to summarise the results.

Whenever available, results were presented as mean ± standard deviation (SD) and organised according to the type of jumping performance variable and, if possible, the competitive level of the participants.

## Results

### Study identification and selection process

**Figure 1:**
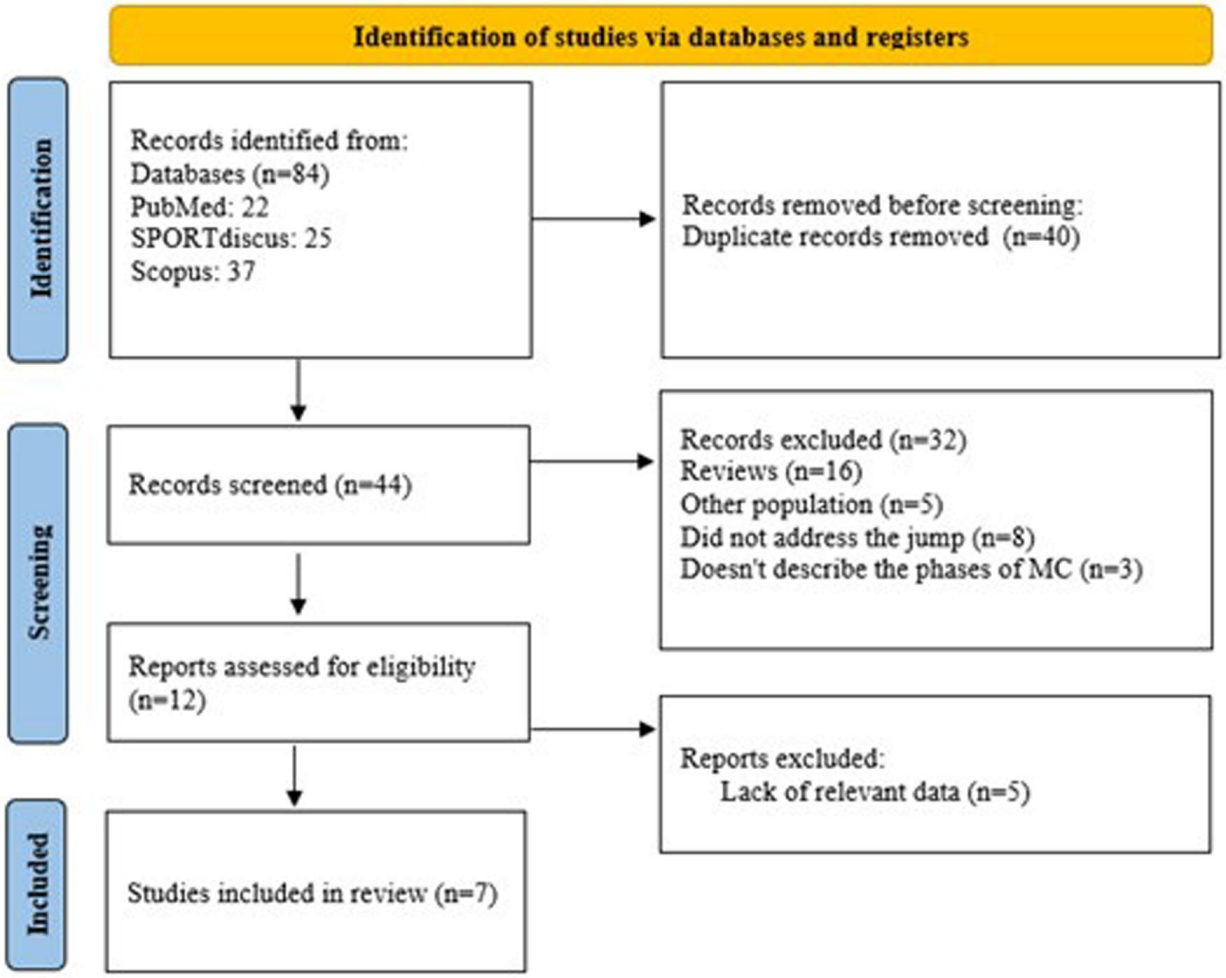
Diagram of the PRISMA method used in the study.

A total of 84 studies were identified through the database search. After removal of duplicates (*n* = 40), 44 records were screened by title and abstract, of which 12 were assessed for full text eligibility. Seven studies met the inclusion criteria and were included in the review (20, 29, 31, 40–43). The complete process of identification, screening, eligibility assessment and inclusion of articles is represented in the PRISMA flowchart (Figure 1).

### General characteristics of the included studies

Due to the methodological heterogeneity of the included studies, only a narrative synthesis was conducted. The main characteristics of the studies are summarised in Table 3.

**Table 3:** Characteristics of the included studies.

Author and date	Competitive level	Methods of determining MC phase	Vertical jump height (CMJ/SJ)	Main results
Sánchez et al. (20)	Regional (*n* = 12)	Mobile application	MP: 27.9 ± 4.49 cm	No significant differences in SJ (*p* > 0.05).
			FP: 28.6 ± 4.35 cm	
			LP: 27.4 ± 5.43 cm	
Julian et al. (29)	Elite (*n* = 9)	Menstruation diary + Hormonal concentrations	EFP: 29.0 ± 3.9 cm	No significant effects of MC on CMJ (*p* > 0.05).
			MLP: 29.6 ± 3.0 cm	
Dasa et al. (40)	Elite team-sport athletes (including football players) (*n* = 29)	Serum hormone analysis	FP: 30.7 ± 2.7 cm	No statistically significant differences in CMJ (*p* > 0.05).
			LP: 31.5 ± 2.8 cm	
Campa et al. (31)	Elite (*n* = 20)	Self-reported	EFP: 28.5 ± 3.5 cm	No significant variation in jump performance was observed (*p* > 0.05).
			OP: 29.3 ± 4.0 cm	
Villaseca-Vicuña et al. (43)	U-17 (*n* = 13)	Calendar application	FP: 28.5 ± 2.59 cm	No significant changes for the two different phases of the MC regarding CMJ (*p* > 0.05).
			LP: 28.9 ± 3.75 cm	
Igonin et al. (42)	Sub-elite (*n* = 11)	Menstruation diary + Hormonal concentrations	EFP: 26.4 ± 3.6 cm	The MC did not significantly influence the results observed in jumping performance (*p* > 0.05).
			LFP: 27.8 ± 4.1 cm	
			MLP: 27.0 ± 3.4 cm	
Aloy et al. (41)	Sub-elite (*n* = 14)	Mobile application	FP: 28.15 ± 4.65 cm	No significant difference in jump height between the MC phases (*p* > 0.05).
			LP: 28.57 ± 4.08 cm	
			OP: 29.28 ± 4.29 cm	

EFP, early follicular phase; LFP, late follicular phase; MLP, mid luteal phase; MP, menstrual phase; OP, ovulatory phase; FP, follicular phase; LP, luteal phase; MC, menstrual cycle; CMJ, countermovement jump; SJ, squat jump; cm, centimetres; *N*, number of subjects.

The included studies comprised women's football players from different competitive levels, including under-17, regional, sub-elite and elite, with a total sample of 108 participants.

Most studies adopted longitudinal designs with intra-individual repeated measures, allowing comparisons across MC phases within the same participants. In most cases, participants served as their own controls. Only one study used a cross-sectional design (43).

The methods used to determine MC phases varied across studies and included menstrual calendars, self-reporting, mobile applications (Mycalendar® and Menstrual Calendar and Cycle® - Period Tracker), and objective hormonal quantification in blood or urine samples. The follicular and luteal phases were the most frequently assessed.

The assessment of vertical jump performance also differed between studies, with the use of force platforms (29, 40), photocell systems (31, 42), mobile application (43), and contact platforms (20, 41).

Sample sizes ranged from 9 to 29 participants. Only one study reported an *a priori* sample size calculation (31) which increases the likelihood of insufficient statistical power in most of the studies included in the analysis.

### Effect of menstrual cycle phase on jumping performance

None of the studies analysed found statistically significant differences in jumping performance between the different MC phases (all *p* > 0.05).

Although some studies have reported slight variations in the average vertical jump height across the phases of the MC, the trend of these differences was not consistent across studies and did not reach statistical significance. This lack of a consistent directional pattern further limits the interpretation of any potential effect.

## Discussion

The aim of this review was to synthesize the available evidence on the potential influence of the MC phase on vertical jump performance in female football players. Results indicate no statistically significant differences in vertical jump performance, either CMJ or SJ across the different MC phases. This pattern was observed at all competitive levels, including elite, sub-elite, U-17, and regional-level players.

These results are consistent with the existing literature. Studies such as those by Julian et al. (29), Dasa et al. (40), Campa et al. (31), Aloy et al. (41), and Villaseca-Vicuña et al. (43) consistently report no significant effects of the MC phase on vertical jump performance. Similarly, Sánchez et al. (20) concluded that speed and explosive strength (vertical and horizontal jumps) are not significantly affected by different MC phase. Igonin et al. (42) also investigated the influence of the MC phases on different neuromuscular capacities in sub-elite female football players and found that jumping capacity was not significantly affected. However, they also observed that short-distance sprint speed (10–30 m) was lower during the early follicular and mid-luteal phases compared to the late follicular phase (near ovulation), suggesting a potential positive effect of increased oestrogen on performance. Also, García-Pinillos et al. (44), found no significant differences in CMJ and sprint performance, but identified significant differences in SJ height (*p* = 0.033, ES = −0.22) in resistance-trained women, revealing superior performance during the follicular phase compared to the ovulatory phase. Recent reviews published on this subject also highlight the existence of inconsistencies in the available evidence and that, overall, the MC seems to have a low magnitude effect, if any, on neuromuscular performance (14), which reinforces the results observed in the present review.

This empirical evidence, however, contrasts with theoretical models suggesting that hormonal fluctuations throughout the MC may influence performance. Oestrogen has been associated with potentially ergogenic effects, including increased neural excitability, improved voluntary activation, and possible anabolic effects on skeletal muscle, particularly during the ovulatory phase (21). On the other hand, progesterone has been associated with potentially negative effects, such as increased body temperature, greater perception of fatigue, and reduced neuromuscular efficiency (17, 22). This theoretical framework supports the hypothesis of better performance during the ovulatory phase and poorer performance during the luteal phase. However, given that jumping ability depends on neuromuscular and structural factors, it is possible that fluctuations in the hormonal profile may not be sufficient to cause any significant, or measurable changes in performance (42).

This apparent discrepancy between theoretical expectations and the findings of the present review can be explained by several factors. First, although it has been demonstrated that hormonal fluctuations influence physiological and neuromuscular mechanisms, these effects may be too small to produce measurable differences in overall performance outcomes, such as vertical jump height. These neuromuscular tasks are highly dependent on multiple determinants, including intermuscular coordination, execution technique, and daily variability (25, 26), which may mask subtle physiological influences. Second, the absence of a consistent directional pattern across studies weakens interpretations based on statistical limitations. Although some studies have reported slight variations between phases, these do not follow a uniform trend (e.g., systematic improvements during the ovulatory phase). Thus, rather than indicating the absence of a trend, the current data suggest that any potential directional pattern cannot yet be characterised reliably, given the methodological heterogeneity, limited hormonal verification, and low statistical power of the available studies (21, 32, 33).

Methodological differences are, in fact, a critical factor. There was considerable heterogeneity in the determination of the MC phase, ranging from subjective methods (self-reported calendars, mobile applications or menstrual tracking diaries) to objective approaches involving hormonal testing. Only a minority of studies used hormonal quantification in blood or urine samples (29, 40, 42), which is considered the gold standard (45). The use of indirect methods, although practical, increases the risk of error in phase classification and may obscure real differences (46, 47). Nevertheless, even among the relatively more robust studies, no consistent pattern has yet emerged; however, the limited number of such studies and their small sample sizes mean that the role of phase-classification accuracy cannot be determined with confidence (45, 48).

Additional heterogeneity was observed in the methods used to assess jumping performance, including force platforms, contact platforms, photoelectric systems, and mobile applications. Force platforms are considered the gold standard for neuromuscular assessment during vertical jump, whereas alternative systems may introduce measurement errors and reduce comparability (49–51). These methodological differences, combined with variability in study design and participant characteristics, likely reduce statistical power and may hinder the detection of small effects. However, the absence of a consistent directional pattern across studies suggests that the observed variability may not be explained solely by statistical limitations (6, 18, 48, 52, 53).

Furthermore, the nature of the tests used may limit the detection of the effects of MC. The CMJ and SJ are short, maximal tasks performed under highly controlled, non-fatigued conditions (25, 26). In contrast, competitive and training environments involve the integration of physiological, psychological, and contextual factors, including fatigue, recovery status, and perceptual responses, which may be more susceptible to fluctuations related to MC (42, 52). In fact, studies report that, although objective performance remains stable, female athletes report a poorer perception of performance, greater fatigue, and lower well-being during specific MC phases (20, 21). Notably, the review by Carmichael et al. (21) consistently demonstrated that female athletes perceive their performance as relatively worse during the follicular and luteal phases, which could be reflected in a greater perception of exertion and lower well-being, associated with a greater perception of fatigue in motor performance.

This dissociation suggests that the potential effects of the MC may manifest more clearly in ecologically valid contexts, such as training and competition, where factors such as accumulated fatigue, competitive stress, and cognitive demands play a decisive role in performance. For example, Igonin et al. (42) observed no significant differences in vertical jump but changes in sprint performance during certain phases, while Sánchez et al. (20) reported significant changes in well-being without an impact on physical performance. These findings reinforce the idea that MC may potentially influence performance readiness more than neuromuscular capacity alone.

From a practical standpoint, the current evidence does not support universal, evidence-based recommendations for modifying training or competition strategies according to MC phase with regard to vertical jump performance. However, monitoring subjective and individual variables may be relevant, especially in high-performance contexts, where small variations can impact competitive performance (54).

Despite these contributions, this review has significant limitations, including the small number of studies, heterogenous sample characteristics, heterogeneity in the determination of the MC phase and assessment protocols and a lack of control for several confounding variables such as training load, sleep, and nutrition (27). These limitations restrict interpretation and generalisability of the findings.

Therefore, future research should prioritize larger sample sizes, standardized protocols, and, above all, the consistent use of hormonal testing to improve the accuracy of classifying the MC phases (45, 48). Additionally, it is essential to recognize that football performance is multifactorial, therefore, the analysis of other performance variables during the MC phases should be included, such as body composition, cardiovascular endurance, or the physical and technical demands during competition.

## Conclusion

The results of this systematic review indicate that the current evidence is insufficient to determine reliably whether MC phase influences vertical jump performance (CMJ and SJ) in female football players, and, if so, in what direction or to what extent. Consequently, the available literature does not support generalisable, evidence-based recommendations for adjusting training according to the phases of the MC with regard to these neuromuscular tasks. However, these results should be interpreted with caution due to the limited number of studies, small sample sizes and substantial methodological heterogeneity.

## Data Availability

The original contributions presented in the study are included in the article/Supplementary Material, further inquiries can be directed to the corresponding author.
