# Supplementary material for: The effects of menstrual cycle phase on jumping performance in women's football players: a systematic review
Source: Front Sports Act Living. 2026 May 29;8:1789611. doi: 10.3389/fspor.2026.1789611 (PMC13260357; doi:10.3389/fspor.2026.1789611)
Supplement: Supplementary file 1 [file Table1.docx]

**The Effects of Menstrual Cycle Phase on Jumping Performance in Women’s Football Players: A Systematic Review**

**Methodological Quality Assessment**

**Table 1** Methodological quality assessment using the adapted Downs and Black checklist

| Study | Item | | | | | | | | | | | | Total Score |
| --- | --- | --- | --- | --- | --- | --- | --- | --- | --- | --- | --- | --- | --- |
|  | 1 | 2 | 3 | 4 | 6 | 7 | 10 | 11 | 12 | 16 | 18 | 20 |  |
| Sánchez et al. (2022) | 1 | 1 | 1 | 1 | 1 | 1 | 1 | 0 | 0 | 1 | 1 | 1 | 10/12 |
| Julian et al. (2017) | 1 | 1 | 1 | 1 | 1 | 1 | 1 | 0 | 0 | 1 | 1 | 1 | 10/12 |
| Dasa et al. (2021) | 1 | 1 | 1 | 1 | 1 | 1 | 1 | 0 | 0 | 1 | 1 | 1 | 10/12 |
| Campa et al. (2022) | 1 | 1 | 1 | 1 | 1 | 1 | 1 | 1 | 0 | 1 | 1 | 1 | 11/12 |
| Villaseca-Vicuña et al. (2024) | 1 | 1 | 1 | 1 | 1 | 1 | 1 | 0 | 0 | 1 | 1 | 1 | 10/12 |
| Igonin et al. (2024) | 1 | 1 | 1 | 1 | 1 | 1 | 1 | 0 | 0 | 1 | 1 | 1 | 10/12 |
| Aloy et al. (2023) | 1 | 1 | 1 | 1 | 1 | 1 | 1 | 0 | 0 | 1 | 1 | 1 | 10/12 |

**Table 2** Item description

| Item | Description | Scoring criteria |
| --- | --- | --- |
| 1 | Is the hypothesis/aim/objective of the study clearly described? | 1 = yes, 0 = no |
| 2 | Are the main outcomes to be measured clearly described in the Introduction or Methods section? | 1 = yes, 0 = no |
| 3 | Are the characteristics of the patients included in the study clearly described? | 1 = yes, 0 = no |
| 4 | Are the interventions of interest clearly described? | 1 = yes, 0 = no |
| 6 | Are the main findings of the study clearly described? | 1 = yes, 0 = no |
| 7 | Does the study provide estimates of the random variability in the data for the main outcomes? | 1 = yes, 0 = no |
| 10 | Have actual probability values been reported (e.g. 0.035 rather than <0.05) for the main outcomes except where the probability value is less than 0.001? | 1 = yes, 0 = no |
| 11 | Were the subjects asked to participate in the study representative of the entire population from which they were recruited? | 1 = yes, 0 = no or unable to determine |
| 12 | Were those subjects who were prepared to participate representative of the entire population from which they were recruited? | 1 = yes, 0 = no or unable to determine |
| 16 | If any of the results of the study were based on “data dredging”, was this made clear? | 1 = yes, 0 = no or unable to determine |
| 18 | Were the statistical tests used to assess the main outcomes appropriate? | 1 = yes, 0 = no or unable to determine |
| 20 | Were the main outcome measures used accurate (valid and reliable)? | 1 = yes, 0 = no or unable to determine |
