# Supplementary material for: The effects of menstrual cycle phase on jumping performance in women's football players: a systematic review
Source: Front Sports Act Living. 2026 May 29;8:1789611. doi: 10.3389/fspor.2026.1789611 (PMC13260357; doi:10.3389/fspor.2026.1789611)
Supplement: Supplementary file 4 [file Datasheet1.docx]

**Search strategy by databases**

PubMed:

("menstrual cycle"[MeSH Terms] OR "menstrual cycle" OR "menstrual phase" OR "hormonal fluctuations" OR "luteal phase" OR "follicular phase" OR "ovulatory phase") AND ("countermovement jump" OR "squat jump" OR "vertical jump" OR "power" OR "neuromuscular" OR "physical performance") AND ("soccer" OR "football").

Filters: English; Publication date from 2015/03/01 to 2025/03/25

Scopus:

(TITLE-ABS-KEY("menstrual cycle" OR "menstrual phase" OR "hormonal fluctuations" OR "luteal phase" OR "follicular phase" OR "ovulatory phase")) AND (TITLE-ABS-KEY("countermovement jump" OR "squat jump" OR "vertical jump" OR "power" OR "neuromuscular" OR "physical performance")) AND (TITLE-ABS-KEY("soccer" OR "football")) AND (LIMIT-TO(LANGUAGE, "English")) AND (PUBYEAR > 2014 AND PUBYEAR < 2026).

SPORTDiscus:

("menstrual cycle" OR "menstrual phase" OR "hormonal fluctuations" OR "luteal phase" OR "follicular phase" OR "ovulatory phase") AND ("countermovement jump" OR "squat jump" OR "vertical jump" OR "power" OR "neuromuscular" OR "physical performance") AND ("soccer" OR "football").

Limiters: English language; Published 2015-2025
